# Molecular Pathogenesis of Cholangiocarcinoma

**DOI:** 10.1186/s12885-019-5391-0

**Published:** 2019-02-28

**Authors:** Peter L. Labib, George Goodchild, Stephen P. Pereira

**Affiliations:** UCL Institute for Liver and Digestive Health, University College London (Royal Free Hospital Campus), Royal Free Hospital, Pond Street, London, NW3 2QG UK

**Keywords:** Cholangiocarcinoma, Aetiology, Pathology, Inflammation, Cholestasis, Molecular pathogenesis, Biomarkers

## Abstract

**Background:**

Cholangiocarcinomas are a heterogeneous group of malignancies arising from a number of cells of origin along the biliary tree. Although most cases in Western countries are sporadic, large population-based studies have identified a number of risk factors. This review summarises the evidence behind reported risk factors and current understanding of the molecular pathogenesis of cholangiocarcinoma, with a focus on inflammation and cholestasis as the driving forces in cholangiocarcinoma development.

**Risk Factors for cholangiocarcinogenesis:**

Cholestatic liver diseases (e.g. primary sclerosing cholangitis and fibropolycystic liver diseases), liver cirrhosis, and biliary stone disease all increase the risk of cholangiocarcinoma. Certain bacterial, viral or parasitic infections such as hepatitis B and C and liver flukes also increase cholangiocarcinoma risk. Other risk factors include inflammatory disorders (such as inflammatory bowel disease and chronic pancreatitis), toxins (e.g. alcohol and tobacco), metabolic conditions (diabetes, obesity and non-alcoholic fatty liver disease) and a number of genetic disorders.

**Molecular pathogenesis of cholangiocarcinoma:**

Regardless of aetiology, most risk factors cause chronic inflammation or cholestasis. Chronic inflammation leads to increased exposure of cholangiocytes to the inflammatory mediators interleukin-6, Tumour Necrosis Factor-ɑ, Cyclo-oxygenase-2 and Wnt, resulting in progressive mutations in tumour suppressor genes, proto-oncogenes and DNA mismatch-repair genes. Accumulating bile acids from cholestasis lead to reduced pH, increased apoptosis and activation of ERK1/2, Akt and NF-κB pathways that encourage cell proliferation, migration and survival. Other mediators upregulated in cholangiocarcinoma include Transforming Growth Factor-β, Vascular Endothelial Growth Factor, Hepatocyte Growth Factor and several microRNAs. Increased expression of the cell surface receptor c-Met, the glucose transporter GLUT-1 and the sodium iodide symporter lead to tumour growth, angiogenesis and cell migration. Stromal changes are also observed, resulting in alterations to the extracellular matrix composition and recruitment of fibroblasts and macrophages that create a microenvironment promoting cell survival, invasion and metastasis.

**Conclusion:**

Regardless of aetiology, most risk factors for cholangiocarcinoma cause chronic inflammation and/or cholestasis, leading to the activation of common intracellular pathways that result in reactive cell proliferation, genetic/epigenetic mutations and cholangiocarcinogenesis. An understanding of the molecular pathogenesis of cholangiocarcinoma is vital when developing new diagnostic biomarkers and targeted therapies for this disease.

## Background

Cholangiocarcinomas are a heterogeneous group of malignancies that occur at any location along the biliary tree [1]. They are anatomically classified as intrahepatic (arising proximal to the second order bile ducts), perihilar (arising between the second order bile ducts and the insertion of the cystic duct into the common bile duct) and distal extrahepatic (arising between the insertion of the cystic duct and the ampulla of Vater) [2]. Although this anatomical classification is widely used, other factors such as tumour growth pattern (mass-forming, periductal infiltrating or intraductal) and the cell of origin (cholangiocytes, peribiliary glands, hepatic progenitor cells or hepatocytes) provide alternative methods of classification that may better predict tumour behaviour [1, 3, 4]. Worldwide, the incidence of intrahepatic cholangiocarcinoma may be increasing whereas perihilar and distal extrahepatic cholangiocarcinomas are decreasing [5]. Incidence rates vary significantly in different countries, probably due to genetic differences and geographical variations in risk factors. In Western Europe, incidences range from 0.45 per 100,000 in Switzerland to 3.36 per 100,000 in Italy [6]. The highest incidence rates are in Asia due to the prevalence of liver fluke infections (e.g. 85 per 100,000 in Northeast Thailand) [5]. Historical under-reporting of cholangiocarcinoma [7], geographical variations in data recording and misclassification of different sub-types means that cancer registry data - and therefore trends in incidence - should be interpreted with caution [8].

The well-described hypothesis of the adenoma-dysplasia-carcinoma sequence observed in many other cancers has not yet been fully characterised in cholangiocarcinoma, due in part to the varying cells of origin that can cause the disease. Intraductal papillary neoplasms of the bile duct demonstrate stepwise progression of oncogenic molecular pathways and increasing dysplasia highly suggestive of an adenoma to carcinoma sequence [9]. Biliary intraepithelial neoplasia, a classification that describes the corresponding molecular and histological changes seen in flat lesions of the bile duct arising from cholangiocytes and peribiliary glands, provides further evidence for such a sequence [10]. This review summarises the risk factors and molecular pathogenesis of cholangiocarcinoma, with a focus on inflammation and cholestasis as the driving forces in cholangiocarcinoma development.

## Risk factors

Although most cases of cholangiocarcinomas in Western countries are considered sporadic [11], there are a number of well-described risk factors (Table 1) [9, 12–24]. It is proposed that many of these risk factors cause chronic inflammation and cholestasis, resulting in a cycle of reactive cell proliferation, genetic and epigenetic mutations and eventual cholangiocarcinogenesis [25].

**Table 1:** Risk factors for cholangiocarcinoma

Risk factors for cholangiocarcinoma	
Cholestatic liver diseases	Primary Sclerosing Cholangitis (PSC)
	Fibropolycystic liver diseases
	Congenital hepatic fibrosis
	Caroli disease
	Choledochal cysts
	Biliary hamartomas
Liver cirrhosis (any aetiology)	
Biliary stone disease	Cholecystolithiasis
	Hepatolithiasis
	Choledocholithiasis
Infections	Liver flukes
	Hepatitis B and C
	Chronic typhoid disease
	Recurrent pyogenic cholangitis
	Human Immunodeficiency Virus (HIV)
Inflammatory disorders	Inflammatory bowel disease
	Chronic pancreatitis
	Gout
	Thyrotoxicosis
Toxins	Alcohol
	Tobacco
	Thorotrast (contrast agent)
	Chemical toxins, e.g. dioxins, vinyl chloride, nitrosamines
Metabolic conditions	Diabetes
	Obesity
	Non-Alcoholic Fatty Liver Disease (NAFLD)
Genetic disorders	Lynch syndrome (Hereditary Non-Polyposis Colorectal Cancer)
	Bile salt transporter protein gene defects
Other	Intraductal Papillary Neoplasms of the Bile duct (IPNB)

### Cholestatic liver diseases

Primary Sclerosing Cholangitis (PSC) is a chronic cholestatic liver disease of unclear aetiology characterised by progressive destruction of the intra- and extrahepatic bile ducts. PSC is strongly associated with inflammatory bowel disease; 60-80% of patients with PSC have a history of ulcerative colitis and 7-21% have a history of Crohn’s disease [26]. Patients with PSC have a 15% lifetime incidence of cholangiocarcinoma (equivalent to a 398-fold increased risk compared to the general population) and up to one third will develop cholangiocarcinoma within a year of being diagnosed with PSC [27, 28]. It is proposed that cholestasis leads to overexposure of cholangiocytes to bile acids that cause abnormal cell proliferation and cholangiocarcinogenesis. Experimental models have shown that bile acids can phosphorylate Epidermal Growth Factor Receptor (EGFR) in cholangiocarcinoma and immortalised cholangiocyte cell lines, leading to cell growth and proliferation [29]. As PSC causes cholestasis, the prolonged exposure of cholangiocytes to bile is likely to be a significant factor in cholangiocarcinogenesis in this disease.

The Fibropolycystic Liver Diseases (FPLD) are a group of conditions characterised by cystic lesions in the liver that are often associated with liver fibrosis and/or renal abnormalities [30]. They arise as a result of abnormal development of the embryonic sheet of biliary precursor cells (the ductal plate) that form the intrahepatic bile ducts and cholangiocytes [31]. FPLD includes congenital hepatic fibrosis, Caroli disease, choledochal cysts and biliary hamartomas [30]. These diseases collectively have a 15% risk of developing cholangiocarcinoma [32]. However, the risk of malignant transformation in FPLD varies depending on the diagnosis; the lifetime risk in patients with choledochal cysts is 15-20% [33], whereas cholangiocarcinogenesis secondary to biliary microhamartomas is rare and it is still debatable as to whether or not it is a true risk factor for the disease [34]. The increased risk is likely to be due to chronic inflammation secondary to impaired biliary drainage, leading to overexposure of cholangiocytes to bile acids and deconjugated carcinogens that were previously conjugated in the liver, reflux of pancreatic secretions into the bile duct, and bacterial contamination [35, 36].

### Liver cirrhosis

Liver cirrhosis is characterised by diffuse fibrosis and nodule formation that occurs as a result of chronic liver injury [37]. The causes of cirrhosis are numerous and include alcohol-associated cirrhosis, non-alcoholic steatohepatitis (NASH), viral hepatitis and autoimmune hepatitis as well as a number of metabolic, congenital and toxic causes [37]. Regardless of aetiology, a number of population-based studies have found cirrhosis to be associated with an increased risk of intrahepatic cholangiocarcinoma [2]. A meta-analysis in 2012 (seven case-control studies, n=339,608) found cirrhosis to have an Odds Ratio (OR) of 22.9 (95% Confidence Interval (CI) 18-2-28.8) for intrahepatic cholangiocarcinoma (ICC) [38]. This may be due to the tissue microenvironment seen in cirrhosis (chronic inflammation, increased cell turnover and progressive fibrosis), which is very similar to the microenvironments seen in a number of other high risk conditions such as PSC [39]. Interestingly, a recent retrospective analysis by Petrick et al. from the US-based Surveillance, Epidemiology, and End Results (SEER) database (2092 ICC, 2981 extrahepatic cholangiocarcinomas (ECC), 323,615 controls) found nonspecific cirrhosis to be associated with both ICC and ECC (ICC OR 8.26, 95% CI 6.83-9.99; ECC OR 3.83, 95% CI 3.05-4.80) [40]. Whilst the liver microenvironment can explain the increased risk in ICC, it is harder to conclude that the same mechanism is responsible for the increased risk of ECC. It may be partly explained by the observation that cirrhosis is linked to lower levels of bile acid excretion, which leads to gut microbiome dysbiosis, a decrease in normal gut microbiata and an increase in pro-inflammatory and pathogenic species which may in turn lead to bacterial contamination of the biliary tree [41, 42]. A confounding factor common to many retrospective analyses is inaccuracy in the anatomical classification of cholangiocarcinoma; many of the cases of ECC are likely to have been perihilar cholangiocarcinomas, which due to their proximity to the liver parenchyma are more likely to be affected by the hepatic microenvironment.

### Biliary stone disease

Gallstones are one of the most common digestive pathologies in the Western world with a prevalence of 10-20% [43]. Usually composed predominantly of cholesterol, they can be found within the gallbladder (cholecystolithiasis), the extrahepatic bile duct (choledocholithiasis) or within the intrahepatic biliary tree (hepatolithiasis). Gallstones are associated with an increased risk of both ICC and ECC [40]. In the aforementioned SEER analysis by Petrick et al., choledocholithiasis was found to confer an OR of 6.94 (95% CI 5.64-8.54) for ICC and 14.22 (95% CI 12.48-16.20) for ECC. Cholecystolithiasis conferred a lower but still significantly increased risk for cholangiocarcinoma (OR 3.93 (95% CI 3.49-4.43) and 5.29 (95% CI 4.83-5.80) for ICC and ECC respectively). An interesting relationship between cholecystectomy and increased risk of cholangiocarcinoma has been observed, although whether or not this is causative remains unclear. A recent systematic review and meta-analysis analysed the data from 4 cohort studies and 12 case-control studies (*n*=220,376 patients with cholecystectomy, 562,392 controls) and found cholecystectomy to be associated with an increased risk for ECC (OR 2.31, 95% CI 1.34-3.28) but not ICC (OR 1.40, 95% CI 0.94-1.87) [44]. One causative mechanism could be the observed change in bile salt composition seen after cholecystectomy where there is a reduction in the circulating pools of primary bile salts but a maintained pool of deoxycholic acid, which is associated with cholangiocyte proliferation (see *Cholestasis and bile acids* below) [29, 45]. It is also possible that the increased risk is secondary to gallstone disease rather than the procedure itself. This is supported by the observation that the increased risk of cholangiocarcinoma reduces to that of the baseline population within ten years of cholecystectomy [46].

Hepatolithiasis, more commonly found in East Asia and associated with liver fluke infections [47] and Caroli disease [48], is also a well-established risk factor for cholangiocarcinoma [49]. A Nationwide multi-institutional cross-sectional survey in Japan in 2006 identified 325 patients with hepatolithiasis, 23 of which having developed cholangiocarcinoma (7%) [50]. The increased risk is thought to be secondary to cholestasis from impaired biliary drainage and inflammation secondary to liver flukes and recurrent bacterial infections [49, 51].

### Chronic infections

Liver fluke infections are endemic in China, Thailand, Korea, Vietnam, Laos, and Cambodia [52]. Cholangiocarcinoma is associated with infection with *Clonorchis sinensis*, *Opisthorchis viverrini* and *Opisthorchis felineus* species, which are usually transmitted through the consumption of raw or undercooked freshwater fish. Mechanical damage from the flukes’ oral and ventral hooks, excreted metabolic products, and granulomatous inflammation surrounding fluke eggs embedded within the periductal tissue all lead to fibrosis and chronic inflammation that results in DNA damage and carcinogenesis [52, 53].

Chronic infection with Hepatitis B and C viruses account for 57% of cases of cirrhosis globally [54]. Several meta-analyses show an increased risk of ICC in both hepatitis B and hepatitis C infection [55–57]. The association with hepatitis C is stronger in regions where hepatitis C is endemic, and likewise for hepatitis B [58]. The largest meta-analysis (13 case-control studies and three cohort studies, *n*=202,135 and *n*=2,655,902 respectively) found hepatitis B to have an OR of 3.17 (95% CI 1.99-5.34) and hepatitis C an OR of 3.42 (95% CI 1.96-5.99) [55].

Chronic typhoid carriers carry a six-fold increase for cholangiocarcinoma [20]. A retrospective analysis of 440 cases of hilar cholangiocarcinoma from a single centre in Egypt (1995-2004) found 52% of patients had a history of typhoid infection, although 54% of patients were also hepatitis C positive, another significant risk factor that could account for part of the increased risk observed [59].

Recurrent Pyogenic Cholangitis (RPC), more commonly encountered in Southeast Asia, is characterised by recurrent primary bacterial infections of the biliary tree resulting in the development of pigment stones and stricturing of the bile ducts [60]. Possible causes are co-infection with liver flukes or breakdown of conjugated bilirubin by bacterial enzymes causing the formation of pigment stones leading to hepatolithiasis, although the evidence for these proposed aetiologies remains sparse [60, 61]. One retrospective study from the US (42 patients, 1986-2005) found 12% of patients developed cholangiocarcinoma, although it is difficult to know if these patients had RPC or hepatolithiasis with recurrent secondary biliary infection. In either case, biliary stone disease associated with recurrent cholangitis is likely to increase the risk of cholangiocarcinoma.

Human Immunodeficiency Virus (HIV) infection may increase the risk of ICC [62]. A U.S. case-control study (625 cases, 90,834 controls) found HIV to have an OR of 5.9 (95% CI 1.8-18.8) [63]. HIV is known to be associated with an increased risk of cholangitis either directly (as part of AIDS cholangiopathy) or indirectly via other opportunistic infections such as cytomegalovirus [63]. It is important to note that this data came from the pre- and early combined antiretroviral therapy era, and multiple relevant confounding diseases with known risk for cholangiocarcinoma were significantly more prevalent in the case population (non-specific cirrhosis, alcoholic liver disease, hepatitis C, diabetes and inflammatory bowel disease). It is therefore possible that the risk of cholangiocarcinoma from HIV is overstated.

Regardless of the pathogen, all of the above infections are characterised by chronicity of infection and sustained inflammation directly or indirectly affecting the biliary tree, leading to mutagenesis, cell proliferation and cancer development.

### Inflammatory disorders

Several inflammatory conditions have been linked to the development of cholangiocarcinoma. Inflammatory bowel disease (IBD) – through its association with PSC – is a risk factor for the development of cholangiocarcinoma. Cholangiocarcinoma occurs at a younger age in IBD patients than in the general population (56 years vs 71 years, respectively). In Western countries, cholangiocarcinoma occurring in patients < 40 years is almost always associated with IBD [64, 65]. PSC-associated cholangiocarcinoma in the presence of IBD appears to follow the dysplasia-carcinoma sequence [66]. The evolution from PSC to cholangiocarcinoma might result from DNA damage by biliary inflammation and bile acids in IBD patients with altered DNA repair functions [67, 68]. Immunosuppression as a result of IBD treatment may also be a contributor in IBD-related carcinogenesis [69].

Two other conditions that may be associated with cholangiocarcinoma are chronic pancreatitis and gout [40]. The mechanisms underlying this may be related to common pathways of chronic inflammation and/or gut microbiome dysbiosis [70–72]. Thyrotoxicosis has been linked to the development of ICC but not ECC (OR 1.25, 95% CI 1.01-1.54) [40]. Untreated hyperthyroidism is known to be associated with abnormal liver function; possible mechanisms include genetic polymorphisms, oxidative stress, and cholestasis secondary to hepatic microcirculatory disorders and damage to hepatocyte and endothelial cell membranes [73–76].

### Toxins

There has been conflicting evidence on the risk of alcohol and tobacco consumption, largely due to the data coming from multiple study designs including population-based, cohort and case-control studies. A recent meta-analysis of 14 cohort studies (*n*=1,515,741 with 410 cases of ICC) found heavy alcohol consumption (≥5 drinks/day) conferred a hazard ratio of 1.68, although the 95%CI was 0.99-2.86 [77]. In contrast, a meta-analysis in 2012 of 11 case-control studies (n=3374 ICC, 394,774 controls) found heavy alcohol consumption (>80g/day or alcoholic liver disease) to confer an OR of 2.81 (95% CI = 1.52-5.21) [38]. This disparity is likely due to the different design methodologies of the included studies; alcohol consumption has been shown to be more strongly associated with liver cancer in case-control studies [78] and cohort studies tend to ask participants about recent alcohol consumption, unlike case-control studies that often estimate lifetime alcohol consumption [77]. Although a meta-analysis in 2013 (six case-control studies, one cohort study) found no difference in cholangiocarcinoma risk between drinkers and non-drinkers (OR 1.09, 95% CI 0.87-1.37), the recent SEER analysis by Petrick et al. found patients with alcohol-related disorders to have an increased risk of cholangiocarcinoma (OR 2.60, 95% CI 2.23-3.04) [40, 79]. Whilst it is likely that alcohol increases the risk of ICC through direct chronic hepatic injury and cirrhosis, the mechanism underlying an increased risk for ECC remains unclear.

Smoking also increases the risk of both ICC (OR 1.46, 95% CI 1.28-1.66) and ECC (OR 1.77, 95% CI 1.59-1.96) [40]. It has been proposed that carcinogenic tobacco compounds damage the biliary epithelium through direct exposure via the circulation [79].

Thorotrast (thorium oxide) was a radiological contrast agent used from 1930-1960 [22]. This compound conferred a 300-fold increased risk of developing cholangiocarcinoma with a latency period of up to 45 years after exposure [80]. Although the mechanism has not been fully elucidated, it is known that Thorotrast is taken up into the reticuloendothelial system and contains an emitter of α-radiation [81]. Combined with its exceptionally long half-life of 400 years, it is likely that chronic exposure to α-radiation lead to direct DNA damage and carcinogenesis.

Exposure to chemical toxins has been linked to outbreaks of cholangiocarcinoma in Italy, West Virginia, and British Columbia, although convincing evidence is lacking [82]. Possible culprits include dioxins, vinyl chloride, nitrosamines, asbestos, the oral contraceptive pill and isoniazid [36, 83, 84].

### Metabolic conditions

Diabetes increases the risk of ICC and ECC [12, 40, 85]. A meta-analysis in 2015 (15 case-control studies and 5 cohort studies, 10,362 patients with cholangiocarcinoma and 351,908 controls) found a combined OR of 1.74 (95% Confidence Interval (CI): 1.62–1.87), although a certain degree of heterogeneity was seen in subgroup analyses of the populations [85]. The recent meta-analysis by Petrick et al. analysed the risk of Type I and Type II diabetes separately and found raised ORs for both ICC and ECC (Type I diabetes OR 1.43 for ICC and 1.30 for ECC, Type II diabetes OR 1.54 for ICC and 1.45 for ECC [40]. All lower values for 95% CI >1.0) [40]. Obesity was also shown to be associated with ICC and ECC, although the OR was greater for ICC (ICC OR 1.42 (95% CI 1.21-1.66), ECC OR 1.17 (95% CI 1.01-1.35)). These findings are consistent with a previous meta-analysis that found obesity to confer an OR of 1.37 (95 % CI 1.22–1.55) for cholangiocarcinoma, although no sub-analysis between ICC and ECC was performed [86].

A new discovery from two recent meta-analyses is the association between Non-Alcoholic Fatty Liver Disease (NAFLD) and cholangiocarcinoma [40, 87]. NAFLD is defined as the presence of hepatic steatosis in the absence of other causes of hepatic fat accumulation (e.g. excessive alcohol consumption, hypothyroidism, etc.) [88]. This can occur in the absence (Non-Alcoholic Fatty Liver, NAFL) or presence (Non-Alcoholic Steatohepatitis, NASH) of inflammation. Non-alcoholic fatty liver disease confers a roughly 3-fold increase in the risk of ICC (OR 3.52, 95% CI 2.87-4.32) and ECC (OR 2.93, 95% CI 2.42–3.55) [40].

There are several proposed causative mechanisms for the inter-related risk factors of diabetes, obesity and NAFLD. Leptin, the hormone responsible for the sensation of satiety, is over-excreted when there is excess adipose tissue and has been shown to enhance cholangiocarcinoma cell growth [89]. Excess adipose tissue causes low-grade systemic inflammation through the release of inflammatory cytokines such as Interleukin-6 (IL-6) and Tumour Necrosis Factor-ɑ (TNFɑ) resulting in chronic hepatic inflammation, cirrhosis and fibrosis [90]. This low grade systemic inflammation is believed to contribute to the onset of insulin resistance and subsequent development of Type II diabetes [40]. The insulin resistance seen in NAFLD, diabetes and obesity results in compensatory systemic hyperinsulinaemia and increased Insulin-like Growth Factor-1 (IGF-1) production in the liver [91, 92]. IGF-1 binding to its receptor (IGF1-R) leads to upregulation of genes involved in cell proliferation and survival [93]. A supporting study for this mechanism by Alvaro et al. found that cholangiocytes from biopsies of normal livers (*n*=10) do not express significant levels of IGF-1 or IGF1-R on immunohistochemical staining, but are both intensely expressed in biopsies of cholangiocarcinoma (*n*=18) [94]. The association between Type I diabetes and cholangiocarcinoma may be explained by the high prevalence of NAFLD (45%) in patients with Type I diabetes [95]. In conclusion, all three conditions are characterised by hepatic steatosis, chronic inflammation, insulin resistance and subsequent upregulation of genes promoting cell turnover, which are all likely to contribute to cholangiocarcinogenesis.

### Genetic diseases

Lynch syndrome (previously known as hereditary non-polyposis colorectal cancer) is an autosomal dominant disorder caused by a germline mutation of one of the four DNA mismatch repair genes. This results in an increased risk of cancers, most commonly colorectal and endometrial cancers but also cancers of the upper gastrointestinal tract, urinary tract and brain. Lifetime risk of a pancreatic or biliary tract cancer is estimated at 2%, although data on cholangiocarcinoma specifically are lacking [96].

A number of congenital abnormalities confer a higher risk for developing cholangiocarcinoma. Defects in genes coding for bile salt transporter proteins (BSEP/ABCB11, FIC1/ATP8B1 and MDR3/ABCB4) cause cholestasis leading to the release of inflammatory cytokines, chronic inflammation and subsequent cholangiocarcinogenesis [97].

### Intraductal Papillary Neoplasms of the Bile Duct (IPNB)

IPNB (previously known as biliary papillomatosis) is a rare disease characterised by the presence of multiple papillary adenomas within the bile ducts. It is associated with hepatolithiasis and liver fluke infection in Asian countries (but not in Western countries) implying both genetic and environmental aetiologies [98]. IPNBs have a high risk of malignant transformation to cholangiocarcinoma, estimated to be as high as 40-80%.

## Pathogenesis

**Fig. 1:**
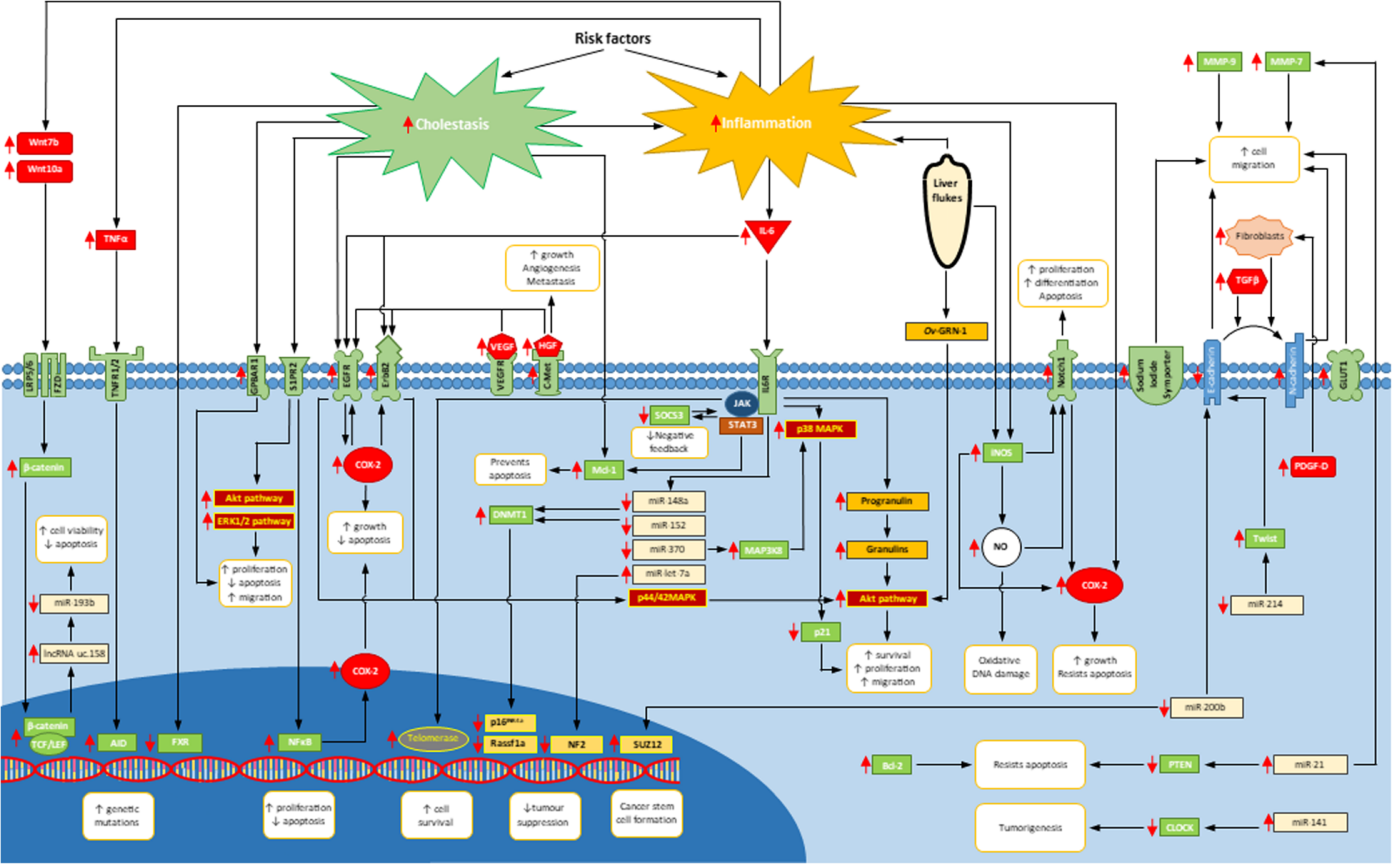
The molecular pathogenesis of cholangiocarcinoma: The majority of risk factors for cholangiocarcinoma cause chronic inflammation and/or cholestasis. Inflammatory mediators such as IL-6 and TNFɑ activate a number of pathways such as JAK-STAT, p38 MAPK and Akt resulting in increased cell growth, survival and proliferation. Macrophages secrete ligands that activate the Wnt/β-catenin pathway, leading to TCF/LEF-mediated gene transcription. Although cholestasis causes inflammation, prolonged exposure of bile acids can have direct cellular effects leading to upregulation of COX-2 and Mcl-1 resulting in resistance to apoptosis. Liver flukes can also have direct effects on cholangiocytes via activation of the Akt pathway and upregulation of iNOS, increasing cell survival and proliferation. A number of microRNAs are up- or downregulated in cholangiocarcinoma. All these alterations lead to well-established oncogenic mechanisms; genetic mutations, increased cell growth, survival, and apoptotic resistance. For a full description of the depicted pathways, please refer to the article text.

Although the above risk factors cover a diverse range of diseases, recurring pathological features in almost all of them are chronic inflammation and/or cholestasis. These two features can provide a unified pathway for the molecular pathogenesis of cholangiocarcinoma by acting on a series of intracellular pathways that encourage carcinogenesis (Fig. 1). Whilst this is unlikely to be a complete model, many of the pathways described below are involved in cholangiocarcinogenesis.

### Inflammation

Inflammation is one of the key factors in cholangiocarcinogenesis. High concentrations of inflammatory mediators cause progressive mutations in tumour suppressor genes, proto-oncogenes and DNA mismatch-repair (MMR) genes, resulting in cell proliferation [99].

The inflammatory cytokine Interleukin-6 (IL-6) affects multiple intracellular pathways that contribute to cholangiocarcinogenesis and can be highly overexpressed in both cultured cholangiocarcinoma cell lines and surgically resected specimens [100]. In normal cholangiocytes, a negative feedback loop for IL-6 exists (IL-6 activates the JAK-STAT pathway, increasing transcription of the cytokine suppressor SOCS3 [99]. In cholangiocarcinoma, epigenetic silencing of SOCS3 is observed, reducing the negative feedback [101]. IL-6 also downregulates specific microRNAs resulting in increased transcription of DNMT1 (an enzyme used to methylate cytosine to alter gene expression) resulting in decreased expression of tumour suppressor genes (see ‘microRNA changes’ below) [102]. By activating STAT3 (a transcription factor in the STAT protein family), IL-6 upregulates Mcl-1 (an apoptosis inhibitor) preventing cell death [103]. IL-6 increases expression of progranulin, a precursor protein for granulins (a family of peptides that regulate cell growth) resulting in activation of the Akt pathway which mediates cell survival, mitosis, migration and angiogenesis [99, 104]. Interestingly, the liver fluke *O. viverrini* secretes a granulin homologue (*Ov*-GRN-1) that can activate the Akt pathway directly resulting in cell proliferation and angiogenesis [105–107]. IL-6 also activates p38 MAPK (a group of protein kinases responsible for cell differentiation and proliferation), resulting in decreased expression of p21 (a mediator of cellular senescence) resulting in mitosis [108]. Lastly, IL-6 reduces telomere shortening by increasing telomerase activity during mitosis, prolonging cell survival [109].

The inflammatory cytokine TNFα causes upregulation of Activation-Induced cytidine Deaminase (AID), an enzyme that creates DNA mutations by converting cytosine to uracil. This results in multiple somatic gene mutations including in tumour suppressor gene p53 and the MYC proto-oncogene [110]. One study showed that AID was barely detectable in biopsies of normal livers (*n*=6) but was present in 80% of cases of PSC (*n*=20) and 93% of cases of cholangiocarcinoma(*n*=30) [110].

Cyclo-Oxygenase-2 (COX-2) is an inflammatory mediator that increases prostaglandin production and is known to be raised in tissue samples of PSC and cholangiocarcinoma [99, 111]. High COX-2 levels can stimulate growth in cholangiocarcinoma, and COX-2 inhibitors can induce apoptosis and inhibit proliferation by decreasing Akt pathway stimulation and activating p21 and other cyclin-dependent kinase inhibitors [112, 113]. COX-2 is partially regulated by inducible nitric oxide synthase (iNOS) which itself is upregulated by inflammatory cytokines. iNOS has been found to be overexpressed in biopsy specimens from patients with advanced (stage III-IV) PSC [114]. The liver fluke *O. viverrini* also expresses iNOS, but the relevance of this has not yet been determined [115]. As well as regulating COX-2, iNOS also increases nitric oxide (NO) production, which results in oxidative DNA damage by affecting DNA repair mechanisms [116]. Both iNOS and NO upregulate Notch1, a transmembrane receptor with a wide variety of functions including cell proliferation, differentiation and apoptosis. Notch1 interacts with COX-2 to make cells more resistant to apoptosis, and has been shown to be upregulated in both intrahepatic and extrahepatic cholangiocarcinoma [117–119].

Recent insights have highlighted the role of macrophages in the activation of the Wnt signalling pathway in cholangiocarcinogenesis. Inflammatory macrophages produce Wnt ligands, which normally have the physiological role of mediating epithelial repair when there is damage to the biliary epithelium [120]. The macrophages upregulate the transcription and production of Wnt7b and Wnt10a, which are excreted and play a paracrine function by binding to the receptor FZD and its co-receptors LRP5/LRP6 on cholangiocytes [120]. Activation of the FZD-LRP5/6 receptor inhibits the intracellular β-catenin degradation complex, leading to an accumulation of β-catenin [121]. β-catenin interacts with the TCF/LEF family of transcription factors in the nucleus, leading to increased cell viability and resistance to apoptosis [122].

### Cholestasis and bile acids

Under normal physiological circumstances, conjugated bile acids can act as ligands for the G Protein-Coupled Bile Acid Receptor 1 (GPBAR1) that affects chloride and bicarbonate excretion, cell proliferation and apoptosis of cholangiocytes [123, 124]. Any obstruction of the flow of bile results in cholestasis and an abnormal accumulation of bile acids within the biliary tree. This results in a decrease in pH leading to enhanced rates of apoptosis [123]. High expression of GPBAR1 has been detected in human-derived samples of cholangiocarcinoma and studies have shown its role as a resistor of apoptosis and promoter of proliferation in cholangiocytes [124, 125]. Conjugated bile acids can also act as ligands for the S1PR2 receptor, leading to activation of the ERK1/2, Akt and Nuclear Factor-Kappa B (NF-κB) pathways resulting in increased COX-2, cell proliferation, migration and survival [126–128]. Excess intracellular bile acids also decrease expression of the nuclear Farnesoid X Receptor (FXR) [129]. Activation of FXR normally results in the excretion of bile acids, and a reduction in FXR causes an intracellular accumulation of bile acids [130]. The bile acid deoxycholic acid increases the survival of Mcl-1 that promotes proliferation, which may be one mechanism by which increased intracellular bile acids promote cell survival [29]. Other specific bile acids (e.g. taurocholic acid) are known to stimulate cholangiocyte proliferation [131], and the bile salt glycochenodeoxycholate has been shown to cause oxidative stress to cholangiocytes and cause subsequent genetic alterations [132]. Conjugated bile acids also activate EGFR leading to increased COX-2 expression and activation of the p38 MAPK and p44/42 MAPK pathways [123, 133], and oxysterols (oxidised cholesterol derivatives found in higher concentrations in cholestatic bile) have also been shown to increase COX-2 mRNA in cholangiocytes [133].

### MicroRNA changes

MicroRNAs (miRNAs) are small non-coding RNA sequences that regulate post-transcriptional gene expression. Multiple miRNAs are upregulated or downregulated in cholangiocarcinoma leading to mitosis, increased cell survival and metastasis [134]. However, many of the studies investigating miRNA expression in cholangiocarcinoma compare cholangiocarcinoma cells with controls, which make it difficult to discern if changes in miRNA expression are part of the process of carcinogenesis or the sequelae of established cholangiocarcinoma [135]. IL-6 has a direct effect on the expression of some miRNAs, and as chronic inflammation likely precedes cholangiocarcinoma, these miRNAs are more likely to be drivers of carcinogenesis. IL-6 increases expression of miR-let-7a, resulting in decreased expression of the tumour suppressor gene NF2 and subsequent STAT3 activation [136]. It also downregulates miR-148a and miR-152 resulting in increased DNMT1 activity leading to methylation of the tumour suppressor genes p16^INK4a^ and Rassf1a [102]. miR-370 is also downregulated by IL-6, leading to increased expression of the oncogene MAP3K8 [137].

The aforementioned upregulation of the Wnt/β-catenin pathway due to the production of Wnt ligands by inflammatory macrophages leads to TCF/LEF gene transcription. This is associated with an increased expression of the long non-coding (lnc) RNA sequence lncRNA uc.158 [122]. lncRNAs, like miRNAs, regulate post-transcriptional gene expression and can also interact with miRNAs [135]. lncRNA uc.158 appears to competitively inhibit miR-193b, which normally has a pro-apoptotic role [122]. This mechanism could explain one of the ways in which activation of the Wnt/β-catenin pathway leads to a reduction in apoptosis.

Many other miRNAs are up- or downregulated in in cholangiocarcinoma, although whether or not many of them are the cause or symptom of cholangiocarcinogenesis remains undetermined. Some example miRNA changes include:

- Decreased miR-200b, leading to an increase oncogene Suz12 and a reduction in E-cadherin expression resulting in cancer stem cell generation and cell migration [138, 139];
- Increased miR-141, decreasing expression of CLOCK, a transcription factor associated with circadian rhythm dysfunction and a number of other malignancies [137, 140, 141];
- Decreased miR-214, leading to increased expression of the transcription factor Twist, reducing E-cadherin levels and subsequent cell migration [142]; and
- Increased miR-21, leading to decreased expression of the tumour suppressor gene PTEN that results in resistance to apoptotic signals [143].

For a more comprehensive review of micro- and other non-coding RNA changes associated with cholangiocarcinoma, see recent reviews by Wangyang et al. (2018) [135] and O’Rourke et al. (2018) [134].

### Other factors affecting spread and invasion

A complex interplay exists between increased levels of extracellular ligands, overexpression of membrane-bound transporters and receptors, and dysregulation of intracellular pathways promoting cell survival and proliferation. Like miRNA changes, it is difficult to say if some of the following observations are a cause or symptom of carcinogenesis due to the design of the experiments that have identified these changes.

The increased levels of cytokine Transforming Growth Factor-β (TGF-β) seen in cholangiocarcinoma causes E-cadherin (a cell-cell adhesion molecule) to switch to N-cadherin resulting in loss of adhesion and an ability to invade [144, 145]. Vascular Endothelial Growth Factor (VEGF), a signal protein key in angiogenesis, is high in both cholangiocarcinoma cell lines and tissue samples *in vitro* [146]. There is evidence that increased VEGF production is driven in part by oestrogens; cholangiocarcinoma cells express oestrogen receptors, can be stimulated to proliferate with 17-β oestradiol, and can have the stimulatory effect of 17-β oestradiol halted with oestrogen receptor antagonists such as tamoxifen [94, 147, 148]. The cell surface receptor tyrosine kinase c-Met, usually only present in progenitor and stem cells for the purpose of organogenesis and wound healing, is abnormally high in cholangiocarcinoma along with its only known ligand Hepatocyte Growth Factor (HGF) leading to tumour growth, angiogenesis and metastasis [149, 150]. VEGF, c-Met, IL-6 and COX-2 all interact with the ErbB receptor kinase family leading to activation of p42/44MAPK (via EGFR and ErB2) and the Akt pathway (via ErB2-driven PI3K activation) [151]. Bcl-2, a potent anti-apoptotic protein, has also been found in high levels in cholangiocarcinoma cell lines [152]. The Sodium Iodide Symporter (NIS), more commonly known for its role in iodide uptake in thyroid follicular cells, is significantly upregulated in cholangiocarcinoma and there is evidence that this leads to increased cell migration and invasion [153, 154]. Increased GLUT-1, a glucose transporter commonly found in several cancers due to increased hypoxia from elevated cell metabolism, is associated with poorer cell differentiation and increased migration and metastasis [155].

Significant stromal changes are also seen in cholangiocarcinoma. Cancer-Associated Fibroblasts (CAFs) in the surrounding stroma produce various factors that promote survival, invasion and metastasis via E- to N-cadherin switching, PI3K-Akt pathway activation and other currently unknown mechanisms [99]. *In vitro* and murine xenograft experiments showed that CAFs express Platelet Derived Growth Factor Receptor β (PDGFR-β), and that cultured cholangiocarcinoma cells secrete the PDGFR-β ligand Platelet Derived Growth Factor-D (PDGF-D) resulting in fibroblast migration and recruitment [156]. Selective blocking of PDGF-D (produced from cholangiocytes) and Rho GTPases (downstream effectors of PDGFR-β activation in CAFs) resulted in reduced CAF migration, supporting this observation. Higher levels of the matrix metalloproteinases MMP-7 and MMP-9 have been observed, resulting in increased extracellular matrix breakdown allowing cells to migrate [157, 158]. Interestingly, the upregulation of MMP-7 appears to be secondary (at least in part) to increased expression of the microRNA miR-21 [158]. Macrophages, whilst playing a role in carcinogenesis through Wnt/β-catenin pathway activation, also appear to play a key role in tumour progression in established cholangiocarcinoma. Cancer stem cells located towards the periphery of the primary tumour appear to secrete a number of molecules (e.g. Interleukin-13, -34 and oesteoactivin) that recruit monocytes and cause them to differentiate into Tumour-Associated Macrophages (TAMs) [159]. A high density of TAMs is associated with tumour invasion, metastasis and worse patient outcomes, suggesting that they are used to create a tumour microenvironment that favours tumour progression [5, 159].

### Genetic and chromosomal factors

Table 2 summarises genetic mutations and polymorphisms associated with cholangiocarcinoma [6, 24, 99, 109, 160–171]. Only a few studies have reported on chromosomal abnormalities in cholangiocarcinoma and the results have been hard to interpret due to the small number of samples and wide genetic variation between the studied population groups. Evidence for gains at 1q, 7p, 8q, 17q, and/or 20q and losses at 1p, 3p, 4q, 6q, 8p, 9pq, 13q, 14q, 17p, 18q and/or 21q have been implicated [162, 172]. Interestingly, genetic variability in cells other than cholangiocytes can be associated with cholangiocarcinoma. For example, Natural killer cells and T-lymphocytes express the receptor NKG2D that plays a role in cell-mediated cytotoxicity and tumour surveillance [161]. One study found that the risk of developing cholangiocarcinoma in patients with PSC varied significantly depending on the NKG2D alleles carried by the patient; some were protective and others more than doubled the risk [173].

**Table 2:** Genetic mutations and polymorphisms associated with cholangiocarcinoma

Gene abbreviation	Gene name	Protein abbreviation	Protein name	Normal function(s)^a^
Congenital mutations/polymorphisms				
ABCB4	ATP Binding Cassette Subfamily B Member 4	MDR3	Multidrug resistance protein 3	Transport of lipids from hepatocytes to bile
ABCB11	ATP Binding Cassette Subfamily B Member 11	BSEP	Bile Salt Exporter Pump	Transport of cholate conjugates from hepatocytes to bile
ABCC2	ATP Binding Cassette Subfamily C Member 2	MRP2	Multidrug resistance-associated protein 2	Transport of endogenous and xenobiotic compounds from hepatocytes to bile
ATP8B1	ATPase Phospholipid Transporting 8B1	FIC1	Familial Intrahepatic Cholestasis type 1	Transmembrane phospholipid transfer
COX-2	Cyclooxygenase 2	COX-2	Cyclooxygenase 2	Inflammatory cytokine
CYP1A2	Cytochrome P450 1A2	CYP1A2	Cytochrome P450 1A2	Xenobiotic metabolism
GST01	Glutathione S-transferase omega-1	GST01	Glutathione S-transferase omega-1	Detoxification of endogenous and xenobiotic compounds
KLRK1	Killer Cell Lectin Like Receptor K1	NKG2D	NKG2-D type II integral membrane protein	Tumour surveillance
MTHFR	Methylenetetrahydrofolate Reductase	MTHFR	5,10-Methylenetetrahydrofolate reductase	DNA methylation
NAT2	N-Acetyltransferase 2	ARY2	Arylamine N-acetyltransferase 2	Drug and carcinogen metabolism
NR1H4	Nuclear Receptor Subfamily 1 Group H Member 4	BAR (FXR)	Bile acid receptor (Farnesoid X receptor)	Negative feedback inhibitor of bile acid synthesis
TYMS	Thymidylate Synthetase	TYMS	Thymidylate synthase	DNA repair
XRCC1	X-Ray Repair Complementing Defective Repair In Chinese Hamster Cells 1	XRCC1	DNA repair protein XRCC1	DNA repair
Acquired mutations				
APC	Adenomatous polyposis coli	APC	Adenomatous polyposis coli	Tumour suppressor
ARID1A	AT-Rich Interaction Domain 1A	ARID1a	AT-rich interactive domain-containing protein 1A	Transcription factor
AXIN1	AXIN1	Axin-1	Axis inhibitor protein 1	Regulates apoptosis
BAP1	BRCA1 Associated Protein 1	BAP1	Ubiquitin carboxyl-terminal hydrolase BAP1	Regulates cell growth
BCL-2	B cell Lymphoma-2	Bcl-2	B-cell lymphoma 2	Regulates apoptosis
BCL2L1	B Cell Lymphoma Like 1	Bcl-xL ^b^	B-cell lymphoma-extra large	Inhibits apoptosis
		Bcl-xS ^b^	B-cell lymphoma-extra small	Promotes apoptosis
BRAF	B Rapidly Accelerated Fibrosarcoma	B-Raf	B-Rapidly Accelerated Fibrosarcoma	Proto-oncogene
BRCA1	Breast Cancer 1	BRCA1	Breast cancer type 1 susceptibility protein	Tumour suppressor and DNA repair
BRCA2	Breast Cancer 2	BRCA2	Breast cancer type 2 susceptibility protein	DNA repair
CCND1	Cyclin D1	CCND1	G1/S-specific cyclin-D1	Regulates cell growth
CDH1	Cadherin 1	E-cadherin	Epithelial cadherin	Tumour suppressor, cell adhesion
CDK6	Cyclin-Dependent Kinase 6	CDK6	Cyclin-Dependent Kinase 6	Controls cell cycle and differentiation
CDKN2A	Cyclin-Dependent Kinase Inhibitor 2A	p16 ^b^	Protein 16	Tumour suppressor
		p14arf ^b^	Protein 14 Alternate Reading Frame	Tumour suppressor
CTNNB1	Catenin Beta 1	Β-catenin	Β-catenin	Proto-oncogene
EGFR (ERBB1)	Epidermal Growth Factor Receptor	EGFR (ErbB-1)	Epidermal Growth Factor Receptor	Proto-oncogene
ERBB2 (HER2)	Avian Erythroblastosis oncogene B2	ErbB-2 (HER2)	Receptor tyrosine-protein kinase erbB-2	Proto-oncogene
FBXW7	F-Box And WD Repeat Domain Containing 7	FBXW7	F-box/WD repeat-containing protein 7	Component of proteasomal protein degradation pathway
FGF19	Fibroblast Growth Factor 19	FGF19	Fibroblast Growth Factor 19	Regulation of bile salt synthesis
FGFR2	Fibroblast Growth Factor Receptor 2	FGFR2	Fibroblast Growth Factor Receptor 2	Cell surface receptor regulating cell proliferation, differentiation, migration and apoptosis
IDH1	Isocitrate dehydrogenase 1	Isocitrate de-hydrogenase 1	Isocitrate dehydrogenase (cytoplasmic)	Glucose metabolism, indirectly mitigates oxidative stress
IDH2	Isocitrate dehydrogenase 2	Isocitrate de-hydrogenase 2	Isocitrate dehydrogenase (mitochondrial)	Glucose metabolism, indirectly mitigates oxidative stress
Keap1	Kelch-like ECH-associated protein 1	KEAP1	Kelch-like ECH-associated protein 1	Prevents Nrf2-driven transcription
KRAS	Kirsten Rat Sarcoma	K-Ras	Kirsten Rat Sarcoma	Proto-oncogene
LTO1	LTO1, ABCE1 maturation factor	LTO1	Protein LTO1 homolog	Ribosome biogenesis
MCL-1	Myeloid Cell Leukaemia 1	Mcl-1 (3 isoforms) ^b^	Induced myeloid leukaemia cell differentiation protein Mcl-1	Isoform 1 resists apoptosis, isoforms 2 & 3 promote apoptosis
MDM2	Mouse Double Minute 2	Mdm2	E3 ubiquitin-protein ligase Mdm2	Proto-oncogene, p53 inhibitor
MYC	Avian myelocytomatosis virus oncogene cellular homolog	Myc	Myc proto-oncogene protein	Proto-oncogene
NF1	Neurofibromin 1	NF1	Neurofibromin	Stimulates Ras activity
PBRM1	Polybromo 1	PBRM1	Protein polybromo-1	Negative regulator of cell proliferation
PIK3CA	Phosphatidylinositol-4,5-Bisphosphate 3-Kinase Catalytic Subunit Alpha	PIK3CA	Phosphatidylinositol 4,5-bisphosphate 3-kinase catalytic subunit alpha isoform	Generates PIP3 that activates signalling cascades for cell growth, survival and motility
PRSS1	Protease, Serine 1	TRY1	Trypsin-1	Serine protease
PRSS2	Protease, Serine 2	TRY2	Trypsin-2	Serine protease
PTEN	Phosphatase And Tensin Homolog	PTEN	Phosphatidylinositol 3,4,5-trisphosphate 3-phosphatase and dual-specificity protein phosphatase PTEN	Tumour suppressor
RAD51AP1	RAD51 Associated Protein 1	RAD51AP1	RAD51 Associated Protein 1	DNA damage repair
RASSF1A	Ras association domain family 1 isoform A	RASSF1A	Ras association domain-containing protein 1 isoform A	Tumour suppressor
ROS1	Reactive Oxygen Species Proto-Oncogene 1, Receptor Tyrosine Kinase	ROS1	Proto-oncogene tyrosine-protein kinase ROS	Epithelial cell differentiation, activation of signal pathways of cell differentiation, proliferation, growth and survival
SMAD4	Small Mothers Against Decapentaplegic 4	SMAD4	Small Mothers Against Decapentaplegic 4	Tumour suppressor, transcription factor
SOCS3	Suppressor Of Cytokine Signaling 3	SOCS3	Suppressor Of Cytokine Signaling 3	Signal transduction inhibitor
TP53	Tumour Protein 53	p53	Protein 53	Tumour suppressor

^a^Relevant to cholangiocarcinoma development. ^b^Through alternate splicing

## Discussion

Even when diagnosed at an early stage, cholangiocarcinoma is an aggressive malignancy with poor patient outcomes. To reduce global mortality from cholangiocarcinoma, efforts must be multifaceted and focus on prevention, early identification of high-risk individuals and prompt diagnosis as well as molecular-based targeted therapies for established disease. Large-scale population studies have provided insight into a number of preventable and modifiable risk factors that could significantly influence disease incidence. The early identification of patients with chronic infections associated with cholangiocarcinoma (e.g. liver fluke infection and typhoid) can allow for early initiation of antibacterial/antiparasitic treatment with a high chance of cure. Although a treatment to eradicate chronic hepatitis B remains elusive, new treatments for hepatitis C can cure many patients [174]. Whilst lifelong treatment can suppress viral replication and prevent cirrhosis, unfortunately access to medication continues to be limited; less than 2% of people with hepatitis B worldwide are on treatment [175]. Global public health initiatives to provide access to medication for hepatitis B and C, and a focus on the modifiable lifestyle factors of alcohol, smoking, and obesity, would have a profound effect on a number of patient outcomes including cholangiocarcinoma incidence. With a global prevalence of 25%, the recent identification of NAFLD as a greater risk factor for cholangiocarcinoma than obesity or diabetes is significant and likely to pose an increasing health burden [176]. Screening patients with PSC for cholangiocarcinoma with regular non-invasive imaging and the tumour marker Carbohydrate Antigen 19-9 (CA 19-9) is done by many centres, although evidence of efficacy of this approach is lacking [177].

As many of the risk factors above cannot be fully eradicated, and the majority of cases of cholangiocarcinoma occur sporadically, an understanding of the molecular pathogenesis of cholangiocarcinoma can allow for the identification of potential early diagnostic biomarkers. For established cholangiocarcinoma, many potential therapeutic targets have been identified in recent years. Drugs have been developed that can target cell surface receptors, their ligands or their intracellular tyrosine kinase components. Example therapies and their respective targets include [160, 178]:

- Intracellular receptor tyrosine kinase blockade by lapatinib (ErbB2), erlotinib and vandetanib (EGFR), sunitinib and cediranib (VEGFR, PDGFR) and ponatinib (Fibroblast Growth Factor Receptor 2 (FGFR2));
- Extracellular antibody blockade by cetuximab and panitumumab (EGFR), brontictuzumab (Notch1) and vanctitumab (FZD7);
- Ligand blockade by bevacizumab (VEGF) and demcizumab (DLL4, the ligand of Notch1) [179].

As many of these receptors have common downstream effectors, other therapeutics have been developed to target their shared intracellular pathways. Both the MAPK/ERK and Akt pathways are activated by the downstream sequelae of cholestasis and inflammation (Fig. 1). Sorafenib, as well as acting as a tyrosine kinase inhibitor on a number of tyrosine kinases including VEGFR-2 and PDGFR, blocks the MAPK/ERK pathway [180]. mTOR, a downstream effector of the Akt pathway, can be targeted using the mTOR kinase inhibitor everolimus [160]. Unfortunately, results from targeted therapies to date have been disappointing. Targeting of EGFR and its downstream pathways by cetuximab, panitumumab and erlotinib has failed to show significant survival benefits in clinical trials [181–183]. A similar lack of response has been observed when targeting VEGF and its downstream pathways by sorafenib or cediranib [184, 185]. As a result, current guidelines only support the use of targeted therapies in the context of clinical trials [186]. Promising future targets include Fibroblast Growth Factor Receptor 2 (FGFR2), Isocitrate Dehydrogenase 1 and 2 (IDH1/2) and Programmed Death Ligand 1 (PD-L1) [8]. Whilst the above results seem discouraging, a significant confounding factor is that many of the earlier trials did not perform molecular profiling of enrolled patients to assess whether or not the target was present in all participants. Future research on targeted therapies will benefit from the wider use of more appropriate study designs, such as basket and umbrella trials.

## Conclusion

Many risk factors have been implicated in cholangiocarcinogenesis, but the evidence supporting each factor is often limited to population-based studies with the inherit limitations of such study designs. Although these risk factors are variable in cause and nature, the majority of them have a common theme of causing chronic inflammation and cholestasis leading to a series of molecular changes that result in reactive cell proliferation, genetic/epigenetic mutations and cancer development. An understanding of the molecular pathogenesis of cholangiocarcinoma is vital when developing new diagnostic biomarkers and targeted therapies to tackle this disease.

## Abbreviations

AID: Activation-Induced cytidine Deaminase
AIDS: Acquired Immune Deficiency Syndrome
AUC: Area Under the Curve
CA 19-9: Carbohydrate Antigen 19-9
CAF: Cancer-Associated Fibroblasts
CI: Confidence Interval
COX-2: Cyclo-Oxygenase-2
DNA: Deoxyribonucleic Acid
ECC: Extrahepatic Cholangiocarcinoma
EGFR: Epidermal Growth Factor Receptor
FGFR2: Fibroblast Growth Factor Receptor 2
FPLD: Fibropolycystic Liver Disease
FXR: Farnesoid X Receptor
GPBAR1: G Protein-Coupled Bile Acid Receptor 1
HGF: Hepatocyte Growth Factor
HIV: Human Immunodeficiency Virus
IBD: Inflammatory Bowel Disease
ICC: Intrahepatic Cholangiocarcinoma
IDH: Isocitrate Dehydrogenase
IGF-1: Insulin-like Growth Factor-1
IGF-1R: Insulin-like Growth Factor-1 Receptor
IL-6: Interleukin-6
iNOS: inducible Nitric Oxide Synthase
IPNB: Intraductal Papillary Neoplasm of the Bile duct
lncRNA: long non-coding RNA
miRNA: micro Ribonucleic Acid
MMP: Matrix Metalloproteinase
MMR: Mismatch Repair
NAFL: Non-Alcoholic Fatty Liver
NAFLD: Non-Alcoholic Fatty Liver Disease
NASH: Non-Alcoholic Steatohepatitis
NF-κB: Nuclear Factor-Kappa B
NIS: Sodium Iodide Symporter
NO: Nitric Oxide
OR: Odds Ratio
PDGF-D: Platelet Derived Growth Factor-D
PDGFR-β: Platelet Derived Growth Factor Receptor-β
PSC: Primary Sclerosing Cholangitis
ROC: Receiver Operating Characteristic
RPC: Recurrent Pyogenic Cholangitis
SEER: Surveillance, Epidemiology, and End Results
TAM: Tumour-Associated Macrophages
TGF-β: Transforming Growth Factor-β
TNF-α: Tumour Necrosis Factor-α
VEGF: Vascular Endothelial Growth Factor
VEGFR: Vascular Endothelial Growth Factor Receptor
